# Innovative Realistic Low-Cost Newborn Chest Drain Manikin

**DOI:** 10.3390/children13040487

**Published:** 2026-03-31

**Authors:** Pankaj Patel, Mayur Prakash, Aldo Perdomo, Stephanie Morakeas, Sandra Warburton, James Elhindi, Dharmesh Shah

**Affiliations:** Newborn Intensive Care Unit, Westmead Hospital, University of Sydney, Sydney, NSW 2145, Australia

**Keywords:** chest drain, manikin, newborn, realistic, low cost

## Abstract

**Highlights:**

**What are the main findings?**

- Chest drain insertion in a newborn infant is a vital skill for neonatal clinicians.
- There are no commercially available models for neonatal chest drain insertion.

**What are the implications of the main findings?**

- It is feasible to build a realistic manikin model for chest drain insertion in newborn infants.

**Abstract:**

**Aim**: Insertion of a chest drain in a newborn infant is a procedure commonly performed in an emergency setting. There are no commercially available neonatal chest drain insertion simulation models. We aim to build an inexpensive and realistic model. **Methods**: A discarded Laerdal Newborn manikin was modified with an internal rib cage, obtained from a decorative Halloween child skeleton. A synthetic silicone layer was used as a subcutaneous tissue and a resealable snack bag as lungs. This cost approximately $110 AUD. Medical staff were invited to use the manikin for chest drain insertion using a Safe-T centesis device. **Results**: Thirty medical officers (50% were registrars and the rest senior medical officers) participated in the study. Fidelity was rated high and there was no difference in the reported aesthetics, tactility, location of anatomy, ease of drainage of air or fluid amongst the registrars, fellows or neonatal consultants. **Conclusions**: It is feasible to build a realistic, high-fidelity manikin for newborn chest drain insertion. The use of a low-cost high-fidelity chest drain model needs to be evaluated in further studies.

## 1. Introduction

Pneumothoraces are common in the neonatal population [1]. The incidence pneumothorax is 1 in 1000 to 5000 term infants. Chest drain insertion is a vital skill for neonatal clinicians to achieve and maintain competency. Most chest drain placements in a newborn infant are urgent or semi-urgent, limiting the ability to teach this skill clinically [2]. Clinicians need the opportunity to learn, practice and maintain their skills in a safe, non-time-pressured environment [3]. High-fidelity simulation-based training is effective in improving knowledge and skill performance post training [4]. Simulation-based training in neonatal procedures would increase competency [5], although skill decay is not uncommon [6]. Low-dose high-frequency simulation-based training would help with improving and retaining skills [7]. There are currently no commercially available neonatal simulation models for chest drain insertion. A model with adequate fidelity that can be constructed by a clinician with local resources would be valuable for teaching and maintaining this skill. A low- to medium-fidelity manikin would assist in promoting realistic training. The use of a higher degree of realism could improve learning in a variety of clinical scenarios [8,9,10]. Locally made manikins do not provide landmarks or the appropriate anatomy of a rib cage. They also do not provide realism in terms of placing a drain going through layers of skin, subcutaneous tissue, intercostal muscle, and pleura. Animal and non-animal models have been reported in the literature for training medical officers in chest drain placement. Ballard et al. used animal models such as a whole chicken for chest tube placement [11]. However, there are concerns about the risk of infection and cleaning of the surroundings. Models using hardware materials like electrical cable wires simulating the rib cage and drawer linen around the rib cage as a skin layer have been used in various models [12,13]. Low-fidelity training resources like a pepper chest and a balloon have been studied for teaching chest drain insertion [14]. Although effective in training, they lacked realism. A high-fidelity manikin will provide a greater degree of physical realism. A newborn manikin with the realism of a rib cage to perform thoracocentesis is currently not available on the market. We aim to build a high-fidelity manikin using locally available resources with a realistic rib cage and landmark identification for performing thoracocentesis.

## 2. Methods

The study was conducted at the Newborn Intensive Care Unit (NICU) at Westmead Hospital, Sydney, Australia.

**Figure 1:**
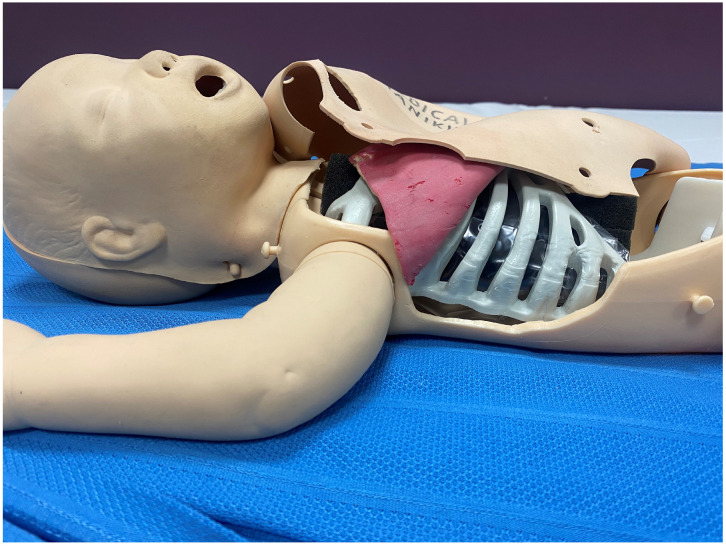
Manikin with a rib cage in situ and subcutaneous layer in the form of the double-layer bowel.

**Figure 2:**
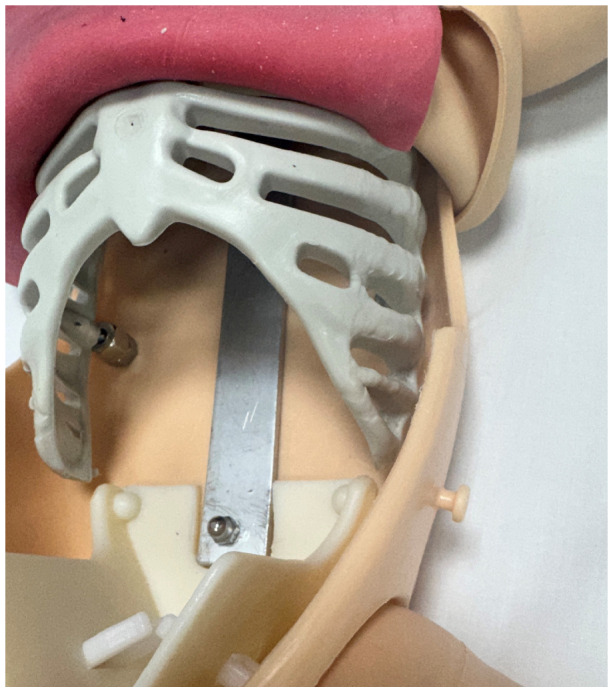
Close-up picture of the manikin showing the rib cage and a resealable snack bag with air.

Model construction: We used an old, non-functional Laerdal Advanced Life Support Training baby manikin, which approximates a three-kilogram newborn (Laerdal Medical Corporation, Stavanger, Norway). The manikin’s original internal mechanics were removed to allow for the placement of a rib cage. A generic life-size Halloween child skeleton purchased online from amazon.com.au (accessed on 10 August 2023), which measures 15 cm in length and 10 cm in breadth ($30 AUD), was used to simulate a rib cage. To facilitate placement within the manikin, the spine attached to the rib cage was removed. This rib cage was screwed to the interior of the manikin. A double-layer bowel [$80 AUD] was used as subcutaneous tissue (Limbs & Things Ltd., Bristol, UK). The simulated bowel layer is a two-layered synthetic silicone bowel model. The bowel layer was glued to the rib cage externally. A single-layer resealable snack bag (5 cents per bag) 15 cm × 10 cm (Hercules^®^ and Click zip^®^, Hercules Industries, Denver, CO, USA) was used to simulate the lungs and was filled with air or water for pleural drainage (Figure 1 and Figure 2).

### 2.1. Recruitment

The study participants were recruited from the NICU at Westmead Hospital. Neonatal consultants, fellows and registrars working in the NICU at Westmead Hospital were eligible for participation in the study. Participation was voluntary. Prospective informed written consent was obtained from all the participants.

### 2.2. Ethics

The study was approved by the Western Sydney Local Health District Human Research Ethics Committee (2023/ETH01568).

### 2.3. Procedure

Participants were provided with a short verbal introduction to the procedure of intercostal drainage. A Safe-T-Centesis 8 Fr Catheter (CareFusion France) was used for thoracocentesis. The participants were shown a video introducing chest drain insertion www.youtube.com/watch?v=2UBK5_e1luk (accessed on 10 August 2023) [15]. The study was performed in the simulation centre at Westmead Hospital on a newborn resuscitaire. Medical officers used gloved hands, and the manikin was covered with a clear plastic drape. The resealable bag was filled with a standard amount air and another bag with water for the drainage of pneumothorax and pleural effusion, respectively, and Styrofoam blocks were used to provide the bulk inside the chest cavity simulating the lungs.

Medical officers were advised to complete the procedure of drainage of air and fluid. The procedure involved correct positioning/placement of the manikin on the resuscitaire, identification of anatomy (sternal bone, intercostal space), incision on the skin, introduction of the chest drain, recognition of pop-off at insertion and aspiration or drainage of the air or fluid. The chest drain was inserted via an incision through the simulated skin, and then the subcutaneous tissue simulated by the bowel layer was punctured, followed by insertion between the ribs (intercostal space) and penetration to the simulated parietal sheet of the pleura (the plastic bag). The time from the incision on the skin to the drainage of air on a successful attempt was recorded.

### 2.4. Evaluation Tool

Medical officers then filled out the evaluation form. A pragmatic 3-point Likert scale [good, average and poor] rating was used for evaluation.

### 2.5. Statistics

Survey responses were summarised according to their sample count and proportion across all respondents. For this analysis, the answers ‘poor’, ‘average’ and ‘good’ were represented by a score of 0, 1 and 2 respectively and compared using a Kruskal–Wallis rank sum test. Sample means and standard deviations were used to describe the responses in each skill level. Analyses were conducted using R Studio Version 4 with a significance level of 0.05.

## 3. Results

Thirty medical officers participated in the study, of which eight were neonatologists. There were seven neonatal fellows and fifteen neonatal registrars. All fellows and neonatologists had more than 5 years of tertiary neonatal experience, whereas half of the registrars had less than 12 months of tertiary NICU experience. None of the registrars with less than 12 months of NICU experience had inserted a chest drain in a newborn infant. Nine participants had inserted a chest drain in the past 6 months. Half of the participants reported having received some simulation-based training in chest drain insertion in the past. Overall, all the participants reported a “good” response to the positioning of the manikin, the anatomy of the rib cage, landmark location, the feel of puncturing the pleura, the ease of drainage and the overall procedure (the response reported was >75% across the group). The participant score was calculated as a mean (±2SD). The aesthetic of the manikin and tactility of tissue were reported to be average by 33% and 27% participants, respectively. The median insertion time from the puncture of skin to the drainage of air and fluid was 45 s (IQR 20–60 s) and 30 s (IQR 15–50 s) respectively. Further analysis comparing the participants (registrars, fellows and neonatologist) showed no difference in the reported aesthetics, tactility, location of anatomy, or ease of drainage of air or fluid within the group (Table 1).

## 4. Discussion

The insertion of a chest drain in a newborn infant is a procedure commonly performed in an emergency setting. It is vital to train medical officers in chest drain insertion to provide care for critically unwell newborn infants. Commercially available trainers for chest drain insertion are limited to adults and paediatric models, and there are none for newborn infants. To address this gap, we built an inexpensive and realistic model using a discarded manikin and readily available products, improving upon past efforts by other researchers.

Zurca et al. built a training model for infant pleural fluid drainage using a hard wire cable to simulate a rib cage with a shelf liner as skin [14]. Our model used similar design with a realistic rib cage, easily identifiable anatomical landmarks and the ability to simulate the drainage of both pleural fluid and air.

Simulation-based learning that incorporates repetitive practice improves learning and supports skill and knowledge retention, especially for critical procedures that appear in emergency situations. A study by Stritzke et al. showed an increase in competency and skill decay with chest tube insertion after 9-12 months [6]. Although skills and skill decay were not assessed in our study, our model provides an opportunity for trainees to undergo regular practice in maintaining these skills. Likewise, recognising and preventing complications with invasive procedures is important. Assessing skill levels in chest drain insertion is an important aspect of training and our model could provide the basis for teaching and supervision [16].

For a model to be viable and have impact in this area, it is important for it to be cost-effective, replicable and biosafe [17]. Our model used a recycled manikin and a rib cage, which eliminated the risk of infection whilst adding realism. There was no statistically significant difference in scoring between medical officers of varied experience. Proceduralists appreciated the realism of our design—particularly its replication of natural anatomical features. The pop-up sensation simulating the puncture of the pleura and entry of the catheter into the cavity along with aspiration of fluid or air as confirmation of proper drain placement was vital in this model. The manikin can be reused multiple times with the only recurring cost being that of the plastic bag to represent the lungs. This manikin serves as a middle ground between the affordability and reproducibility of the design outlined by Winckworth et al. and the fidelity and accuracy of that developed by Zurca et al. [13,14]. Reusing hospital material and recycling discarded manikins add value to the model.

3D-printed models have increasingly been used for simulation [18]. Moron et al. constructed and validated a low-cost simulator for teaching thoracocentesis in an adult manikin [19]. They used biological material and evaluated the feasibility of a low-cost simulator for training in procedural skills like tube thoracocentesis.

Our model replicates the adult chest drain model by Young et al., in which they used a Halloween skeleton, a plastic dress-form torso and a yoga mat with sandwich bags as pleura [20]. Our model has improvements on the fidelity of the pleural cavity. Tatli et al. studied 63 interns simulating chest drain insertion in a manikin and reported an 85% success rate with high skill scores [21]. Although we did not study individual skills of the participants, we were able to demonstrate high overall procedural scores for fidelity, anatomical locations and success rates. We built upon the methodology of Tatli et al. by including fellows and consultants in our study as a way of providing a more robust assessment of the objective features of the manikin as assessed by more experienced doctors. Although adult manikin models providing the realism of a chest drain are commercially available, no newborn models are available to best of our knowledge. Our model demonstrates the feasibility of building a low-cost manikin providing realism for newborn chest drain.

## 5. Limitations

Our study had various limitations. This was a pragmatic study with a small sample size for building a manikin and assessing the fidelity. Our model did not study skills transfer to clinical performance nor validation of the manikin. There was a need to change bags after each procedure, and it would be desirable to have a resealable bag to simulate the pleura. The texture of the skin was not realistic, and the effect of spontaneous breathing was not evaluated. Similarly, the risk of injury to the intercostal vessels could not be evaluated. The 3-point Likert scale was used for this model, and further studies using this manikin could use a more detailed and validated assessment tool including inter-rater reliability and skill decay.

## 6. Strength

In our model, anatomical landmarks like the intercostal space and skin and muscle were identifiable. There was a pop-off sound with the insertion of the catheter in the pleural cavity which provided realism. This is a low-cost manikin and could be replicable widely.

## 7. Conclusions

It is feasible to build a realistic procedural manikin for chest drain insertion in newborns. Further studies are needed to assess inter-rater reliability and skill decay.

## Figures and Tables

**Table 1:** Evaluation of the manikin amongst participants in draining air and fluid.

Feature	Registrar (15)	Fellow (7)	Consultant (8)	Registrar (15)	Fellow (7)	Consultant (8)
	Air Mean (SD)			Fluid Mean (SD)		
Aesthetics	1.5 (0.5)	1.4 (0.8)	1.9 (0.4)	1.5 (0.5)	1.4 (0.8)	1.8 (0.7)
Positioning	1.9 (0.3)	1.9 (0.4)	2.0 (0)	1.9 (0.3)	1.9 (0.4)	2.0 (0)
Tactility of tissues	1.5 (0.6)	1.7 (0.8)	1.6 (0.5)	1.7 (0.5)	1.7 (0.8)	1.8 (0.5)
Anatomy of rib cage	1.9 (0.3)	1.4 (0.8)	1.6 (0.5)	1.9 (0.3)	1.6 (0.8)	1.5 (0.5)
Location of landmarks	1.9 (0.4)	1.7 (0.5)	1.6 (0.5)	1.9 (0.3)	1.9 (0.4)	1.6 (0.5)
Insertion and puncturing the pleura (pop-off)	1.7 (0.6)	1.6 (0.8)	1.9 (0.4)	1.9 (0.4)	1.7 (0.8)	1.6 (0.7)
Ease of draining air	1.8 (0.4)	2.0 (0)	1.8 (0.5)	1.9 (0.4)	2.0 (0)	1.9 (0.4)
Overall procedure	1.9 (0.3)	1.9 (0.4)	1.9 (0.4)	1.9 (0.5)	1.9 (0.4)	1.8 (0.5)
Duration (s) Median (IQR)	41 (26)	28 (16)	62 (37)	31 (21)	43 (26)	37 (31)

SD = ±2 standard deviation; IQR = Interquartile Range.

## Data Availability

The original contributions presented in this study are included in the article. Further inquiries can be directed to the corresponding author.

## Abbreviation

NICU	Newborn Intensive Care Unit
